# Calcium overload induces reactive oxygen species to enhance glioblastoma radiosensitivity

**DOI:** 10.4103/mgr.MEDGASRES-D-26-00022

**Published:** 2026-06-05

**Authors:** Xin Lai, Yijun Lu, Jie Li, Lingling Liu, Shuanghu Yuan, Junchao Qian

**Affiliations:** 1 First Clinical Medical College, Anhui University of Science and Technology, Huainan, Anhui Province, China; 2 Anhui Province Key Laboratory of Medical Physics and Technology, Institute of Health and Medical Technology, Hefei Institutes of Physical Science, Chinese Academy of Sciences, Hefei, Anhui Province, China; 3 Department of Radiation Oncology, The First Affiliated Hospital of USTC, Division of Life Sciences and Medicine, University of Science and Technology of China, Hefei, Anhui Province, China; 4 Department of Radiation Oncology, Anhui Provincial Cancer Hospital, Hefei, Anhui Province, China

**Keywords:** apoptosis, calcium homeostasis, calcium overload, endoplasmic reticulum stress, ionomycin, oxidative stress, protein misfolding, radiosensitization, treatment resistance, unfolded protein response

## Abstract

Radiotherapy is the primary treatment of glioblastoma, but its efficacy is often limited by tumor cell resistance to radiation. Radiotherapy mainly has its cytotoxic effect through the formation of reactive oxygen species and the damage to DNA. Nevertheless, to overcome the effects of reactive oxygen species-mediated oxidative damage and endoplasmic reticulum stress, tumor cells may activate the repair mechanisms to stress and improve antioxidant defenses. This study innovatively proposes the use of the calcium ionophore ionomycin as a radiosensitizer in glioblastoma. We used U87MG and U251 cell lines as well as subcutaneous xenograft mice as models, and conducted a combined intervention of ionomycin and radiotherapy. Mechanistically, ionomycin selectively disrupted endoplasmic reticulum calcium homeostasis, inducing severe and sustained endoplasmic reticulum stress. When combined with radiotherapy, this led to a marked surge in intracellular reactive oxygen species, and significantly enhanced apoptosis. *In vitro*, the combination treatment synergistically reduced cell viability, clonogenicity, and proliferation compared with either monotherapy. *In vivo*, ionomycin combined with radiotherapy substantially suppressed tumor growth and increased intratumoral reactive oxygen species levels and apoptosis. These findings indicate that ionomycin converts repairable adaptive stress into irreversible lethal damage by amplifying reactive oxygen species through an endoplasmic reticulum stress-reactive oxygen species vicious cycle, thereby overcoming antioxidant defenses and enhancing glioblastoma radiosensitivity.

## Graphical abstract

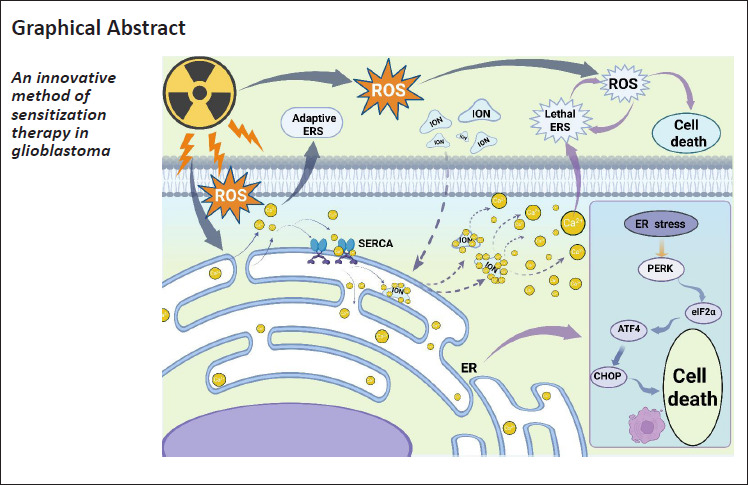

## Introduction

The primary central nervous system malignant tumor that occurs most frequently and is the most aggressive is glioblastoma (GBM).^1^,^2^ Although there have been improved methods of diagnosis and treatment over the past few years, the prognosis of a patient with GBM is poor, with a short median survival time.^3^,^4^ Ionizing radiation (IR) is a key component of the standard treatment regimen for GBM, and its effect is mainly based on causing damage to DNA and the formation of reactive oxygen species (ROS) in cancerous cells.^5^,^6^ The burst of ROS is a core initiating factor triggering downstream cellular stress and death programs.^7^ Nevertheless, tumor cells can efficiently clear ROS through the strengthening of anti-oxidant defenses and pro-survival signal transduction, which undermine their lethal action and results in therapeutic resistance.^8^ Therefore, exploring strategies that can effectively induce and sustain a ROS burst exceeding the cell’s clearance capacity, overcome tumor adaptive tolerance, and synergize with IR has become a crucial research direction for improving GBM treatment efficacy.

Endoplasmic reticulum stress (ERS) and intracellular ROS generation have a two-way regulatory interaction.^9^,^10^ ERS may cause protein folding disorders and an imbalance in calcium homeostasis, which in turn causes mitochondrial dysfunction with excessive production of secondary ROS.^11^,^12^,^13^ On the other hand, acute increases in the levels of ROS can then target the endoplasmic reticulum, worsening its oxidative stress and disturbance of functionality, creating a vicious cycle.^14^ A previous study indicates that IR, per se, may cause an endoplasmic reticulum calcium leakage, which may cause mild ERS with low-ROS increment.^15^ However, compensatory actions usually counter the effects of this strain, including the endoplasmic reticulum calcium pump (sarco/endoplasmic reticulum calcium ATPase, SERCA), and ERS intensity and extent of ROS is not enough to exceed the repair threshold in the cell.^16^,^17^,^18^ Thus, understanding possible strategies of jointly enhancing and sustaining the ERS-ROS vicious cycle throughout IR is instrumental in breaking radioresistance in GBM.

Ionomycin (ION), being a calcium ionophore, may use calcium ions across membranes and is frequently employed as a tool molecule to regulate the intracellular calcium concentration.^19^,^20^,^21^ The study aimed to offer a novel combination treatment concept to overcome the GBM radioresistance, and experimental evidence for a deeper understanding of the central role of the calcium homeostasis–ERS–ROS axis in determining tumor cell fate.

## Methods

### Animal experiments

**Figure 1:**
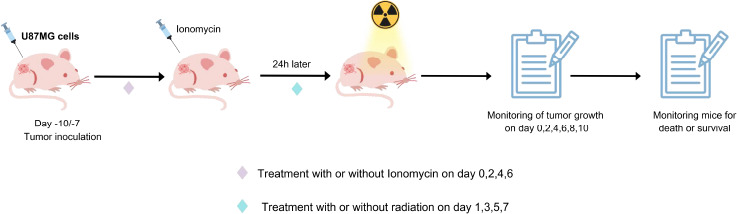
Flowchart of *in vivo* experiments. Created using Procreate (version 5.4.2) and Canva.

The *in vivo* effects of ION on GBM growth and apoptosis were investigated through subcutaneous tumor implantation in nude mice (**Figure 1**). Five-week-old male BALB/c nude mice (weighing about 20 g) were purchased from Zhejiang VTLH Laboratory Animal Technology Co., Ltd. (Zhejiang, China, license No. SCXK (Zhe) 2024-0001). The research protocol and all subsequent procedures involving animal care and handling were approved by the Animal Ethics Committee of the Hefei Institutes of Physical Science, Chinese Academy of Sciences on April 8, 2025 (approval No. DWLL(E)-2025-28). All experiments were designed and reported according to the Animal Research: Reporting of *In Vivo* Experiments (ARRIVE) guidelines.^22^

U87MG GBM cells (a human GBM cell line; Servicebio, Wuhan, China; STCC11005P) were extracted and counted, with cell concentration adjusted to 5 × 10^7^/mL. A 100 μL cell suspension was injected subcutaneously into the upper right inguinal region of nude mice. 7–10 days after tumor formation confirmation, BALB/c nude mice were randomly divided into four groups: model, IR, ION, and IR + ION groups (*n* = 4/group). Mice in the IR and IR + ION groups were administered a total radiation dose of 20 Gy, with four doses of 5 Gy each, spaced 1 day apart. Mice in the ION and IR + ION groups received 3 mg/kg ION via tail vein injection, and tail vein injection was performed at 1 day before each irradiation session (**Figure 1**). Tumor volumes were measured every 2 days using a digital caliper and calculated as volume = (length × width²)/2. At the end of the experiment, tumors were excised, photographed, and weighed.

### Cell culture

**Figure 2:**
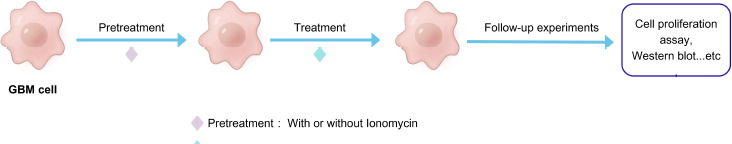
Flowchart of *in vitro* experiments. Created using Procreate (version 5.4.2) and Canva.

Human GBM cell line U251 was purchased from Servicebio (STCC11007P) and authenticated by short tandem repeat analysis (**Figure 2**). U87MG and U251 cells were cultured in Dulbecco’s modified Eagle medium (Biosharp, Hefei, China, BL304A) supplemented with 10% fetal bovine serum (Clark, Richmond, VA, USA, FB25015) and 1% penicillin/streptomycin (Hyclone, Logan, UT, USA, SV30010) at 37°C with 5% CO_2_.

### Cell irradiation and drug treatment

U87MG and U251 cells were incubated with 10 mM N-acetyl-L-cysteine (NAC; a ROS scavenger; Aladdin, Shanghai, China, 616-91-1; 10 mM in dimethyl sulfoxide) for 30 minutes. After that, cells were exposed to different radiation doses. In the clonogenic assay, radiation doses of 0, 4, 8, and 10 Gy were used; in other cell experiments, a radiation dose of 10 Gy was applied. All cells were incubated with the drug prior to irradiation; the control IR group was not treated with the drug but underwent radiation therapy under the same conditions as the other groups.) using a 6 MV medical linear accelerator (Elekta Axesse, Stockholm, Sweden). For combination treatment, cells were pretreated with ION.

### Cell viability assay

U87MG and U251 cells were seeded in 96-well plates. For inhibitor studies, cells were pretreated with ISRIB (a protein kinase R-like endoplasmic reticulum kinase (PERK) pathway inhibitor; Beyotime, Shanghai, China, SC4332) for 1 hour before 4–6-hour incubation of 4 µM ION (YeSen, Shanghai, China, 50402ES03), followed by 10 Gy X-ray irradiation. After 24 hours, cell viability was measured using the cell counting kit-8 assay (YeSen, 40203ES60). Absorbance was measured at 450 nm using a microplate reader (Thermo Fisher Scientific, Waltham, MA, USA, Multiskan GO). Cell viability was expressed as the percentage relative to the control group (set as 100%).

### Cell proliferation assay

U87MG cells were seeded in 96-well plates, treated with 4 µM ION for 2 hours, and irradiated (10 Gy). Cell proliferation was monitored at 0, 24, 48, and 72 hours post-irradiation using the cell counting kit-8 assay. The absorbance was measured at 450 nm using a microplate reader. Cell proliferation was expressed as the normalized relative cell survival rate within each treatment group relative to the 0-hour time point.

### Cell clonogenic assay

U87MG cells were counted and seeded into 6-well plates at a density of 1 × 10³ cells per well. Based on the presence or absence of ION and IR treatment, the cells were divided into groups. Radiation doses were set at 0, 4, 8, and 10 Gy. The culture medium was replaced twice per week, and cells were cultured for 10–14 days until colonies became visible under a microscope (Leica, Wetzlar, Germany, DM6B). Following colony formation, the medium was aspirated, and colonies were washed twice with phosphate-buffered saline (PBS), fixed with 4% paraformaldehyde for 30 minutes, and washed again with PBS. Subsequently, colonies were stained with 10% crystal violet (BBI, Shanghai, China, E607309-0100) for 15 minutes, rinsed with distilled water, and air-dried. Finally, colonies were counted and quantified using ImageJ software (1.44p; National Institutes of Health, Bethesda, MD, USA).^23^ The clonogenic rate (%) was calculated as: (number of colonies in the treatment group/number of colonies in the control group) × 100.

### Live/dead staining

U87MG and U251 cells were seeded at 1 × 10^5^ cells/well in 12-well plates. After 24 hours, the NAC group was pretreated for 1 hour before adding 4 μM ION for 4–6 hours, followed by 10 Gy irradiation and 24 hours of incubation. Cells were washed with PBS and stained with 2 μM Calcein-AM and 4.5 μM PI (Beyotime, C2015S) for 30 minutes, washed again, and imaged under a fluorescence microscope.

### Apoptosis analysis

U87MG cells were seeded at 5 × 10^5^ cells/well in 6-well plates. After 24 hours, the NAC group was pretreated for 30 minutes before ION and IR addition. After treatment, cells were digested with ethylenediaminetetraacetic acid-free trypsin, collected by centrifugation (4°C, 5 minutes), and washed twice with PBS. Cells were resuspended in 100 μL 1× binding buffer, stained with 5 μL Annexin V-FITC and 10 μL PI for 10–15 minutes, diluted with 400 μL Annexin V-FITC binding buffer, and analyzed by flow cytometry (BD, Franklin Lakes, NJ, USA, FACSCanto II).

### Intracellular calcium detection

Intracellular Ca²^+^ levels were measured using the Fluo-4 AM calcium kit (Beyotime, S1061S). U87MG and U251 cells were loaded with 0.2 μL Fluo-4 AM per sample at 37°C for 30 minutes. Subsequent steps were performed in the dark, and fluorescence was immediately observed under a fluorescence microscope.

### Western blot analysis

U87MG and U251 cells were lysed, and protein concentration was measured using a bicinchoninic acid kit (Beyotime, P0012). Proteins were separated by 10% sodium dodecyl sulfate polyacrylamide gel electrophoresis, transferred to polyvinylidene fluoride membranes, blocked with 5% skim milk for 1 hour, and incubated overnight at 4°C with primary antibodies: PERK (1:1000, mouse, Wuhan Sanying, Wuhan, China, Cat# 68482-1-Ig), phospho-PERK (p-PERK; 1:1000, rabbit, MedChemExpress, Monmouth Junction, NJ, USA, Cat# HY-P81190, RRID:AB_3103093), glucose-regulated protein 78 (GRP78; 1:1000, rabbit, Wuhan Sanying, Cat# 11587-1-AP), eukaryotic translation initiation factor 2α (eIF2α; 1:1000, rabbit, Wuhan Sanying, Cat# 11170-1-AP), phospho-eIF2α (p-eIF2α; 1:1000, rabbit, Wuhan Sanying, Cat# 28740-1-AP), activating transcription factor 4 (ATF4; 1:1000, mouse, Wuhan Sanying, Cat# 60035-1-Ig), C/EBP homologous protein (CHOP; 1:1000, rabbit, Wuhan Sanying, Cat# 15204-1-AP), and β-tubulin (loading control; 1:1000, rabbit, Wuhan Sanying, Cat# 10094-1-AP). Membranes were washed, incubated with HRP conjugate Goat anti-Mouse IgG (H&L) or HRP conjugate Goat Anti-Rabbit IgG H&L (1:10,000, Zen Bio, Durham, NC, USA, Cat# 511103/511203, RRID:AB_2893489/AB_2927753) at room temperature for 1 hour, and developed with an electrochemiluminescence kit (Servicebio, G2074). Blots were imaged using a chemiluminescence imaging system (5200Multi, Thermo Fisher Scientific), and band intensities were quantified using ImageJ software. The expression level of each target protein was normalized to β-tubulin, and data were expressed as a fold change relative to the control group.

### Intracellular reactive oxygen species staining

Intracellular ROS was detected using the 2′,7-dichlorodihydrofluorescein diacetate (DCFH-DA) probe (Aladdin, 4091-99-0). U87MG and U251 cells (100,000/well, 12-well plate) were treated with 4 μM ION for 4–6 hours, irradiated (10 Gy), and incubated for 24 hours. Cells were washed with PBS, stained with DCFH-DA (1:1000) for 30 minutes, washed again, and observed under a fluorescence microscope.

### Quantitative polymerase chain reaction

Total RNA was extracted from U87MG cells using the TransZol Up Plus RNA Kit (Transgen Biotech, Beijing, China, ER501-01). Complementary DNA was synthesized using HiScript® II Q Select RT SuperMix (Vazyme, Nanjing, China, R233). Quantitative polymerase chain reaction was performed with ChamQ Universal SYBR qPCR Master Mix (Vazyme, Q711) on a Roche LightCycler 96 (Little Falls, NJ, USA). Glyceraldehyde-3-phosphate dehydrogenase served as the endogenous control. Primers were synthesized by Generalbiol (Chuzhou, China) and listed in **Additional Table 1**.

**Additional Table 1:** qPCR primer sequences

Gene	Species	Primer sequence
*GAPDH*	Human	Forward: 5'-ACA ACT TTG GTA TCG TGG AAG G-3' Reverse: 3'-GCC ATC ACG CCA CAG TTT C-5'
*PERK*	Human	Forward: 5'-GGA AAC GAG AGC CGG ATT TAT T-3' Reverse: 3'-ACT ATC TCC ATT ATG CCA GCT TC-5'
*ATF4*	Human	Forward: 5'-CTC CGG GAC AGA TTG GAT GTT-3' Reverse: 3'-CGC TGC TTA TTA CTC TCC TGG AC-5'
*CHOP*	Human	Forward: 5'-GGA AAC AGA GTG CTC ATT CCC-3' Reverse: 3'-CTC CTT GAG CCG TTC ATT CTC-5'

ATF4: Activating transcription factor 4; CHOP: C/EBP homologous protein; GAPDH: glyceraldehyde-3-phosphate dehydrogenase; PERK: protein kinase R-like endoplasmic reticulum kinase; qPCR: quantitative polymerase chain reaction.

### Hematoxylin and eosin staining

Mice were anesthetized with avertin (0.2 mL/10 g; Aladdin, R1511179) and euthanized by cervical dislocation. The tumor tissues were fixed in 4% paraformaldehyde overnight, dehydrated in 30% sucrose, sectioned (20 μm), stained with hematoxylin (Biosharp, BL702B) for 10 minutes and eosin (Biosharp, BL703B) for 2 minutes, differentiated in 1% acidic ethanol, mounted, and examined under a fluorescence microscope.

### Terminal transferase dUTP nick end labeling staining

The tumor sections were incubated with terminal transferase dUTP nick end labeling kit (Heruibio, HRX1204) for 1 hour at 37°C, washed, incubated with streptavidin-tetramethylrhodamine isothiocyanate (Heruibio, Fuzhou, China, HRX1204) for 30 minutes, counterstained with 4′,6-diamidino-2-phenylindole (Servicebio, G1012), and imaged by fluorescence microscopy.

### Tissue reactive oxygen species staining

Fresh tumor tissues were embedded in optical coherence tomography, sectioned, stained with DCFH-DA for ROS detection, counterstained with 4′,6-diamidino-2-phenylindole, and imaged under a fluorescence microscope.

### Statistical analysis

This study was based on preliminary experimental results and used an appropriate statistical power analysis to determine the sample size per group. All statistical analyses were performed using GraphPad Prism 9.3 software (GraphPad Software, Boston, MA, USA, www.graphpad.com). The unpaired *t*-test was used to compare differences between two groups, while one-way analysis of variance was employed to compare differences among multiple groups, followed by Dunnett’s test for multiple comparisons. Data are expressed as mean ± standard deviation (SD). *P* < 0.05 was considered statistically significant.

## Results

### Ionomycin synergizes with radiotherapy to enhance anti-tumor effects *in vitro*

**Figure 3:**
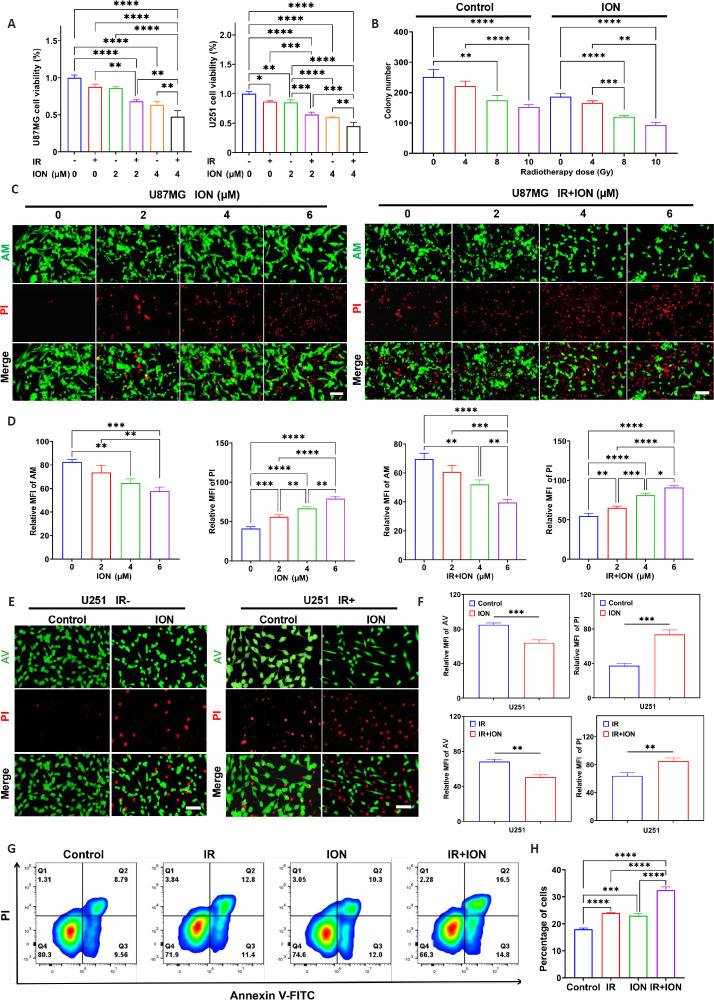
ION combined with IR inhibits GBM cell viability and induces apoptosis. (A) Cell viability of U87MG and U251 cells treated with varying doses of ION and/or IR was assessed by CCK-8 assay. (B) Quantitative analysis of clonogenic assay results. (C, D) U87MG cell viability was assessed using live/dead assay (C), and quantitative analysis of MFI was performed (D). (E, F) ION combined with IR induce cell death in U251 cells. Scale bars: 200 µm. (G, H) Apoptosis in U87MG cells was detected by flow cytometry (AV/PI staining). Data are expressed as mean ± SD (*n* = 3). *P < 0.05, **P < 0.01, ***P < 0.001, ****P < 0.0001 (one-way analysis of variance followed by Dunnett’s test (A, B, D, H) or unpaired *t*-test (F)). AV: Annexin V; CCK-8: cell counting kit-8; GBM: glioblastoma; ION: ionomycin; IR: ionizing radiation; MFI: mean fluorescence intensity; PI: propidium iodide.

To evaluate the radiosensitizing effect of ION, the cell counting kit-8 assay showed that ION alone exhibited dose-dependent cytotoxicity against U87MG and U251 cells (half-maximal inhibitory concentration ≈ 4.163 μM) (**Additional Figure 1A** and **B**). IR combined with ION treatment proved much more effective in inhibiting cell viability than all other treatments (**Figure 3A**). This was further established through clonogenic and cell proliferation assays, revealing that the combination treatment nearly inhibited long-term clonogenic and proliferative ability of the cells (**Figure 3B** and **Additional Figure 1C** and **D**). The Calcein-AM/PI dual staining showed IR combined with ION treatment was significantly stronger in enhancing the percentage of cell death (**Figure 3C–F**). In addition, apoptosis through the supplementation of Annexin V/PI flow cytometry displayed a higher rate of apoptosis in the combined treatment group than the monotherapy group (**Figure 3G** and **H**). Such findings indicate that ION can promote the effect of IR killing, which may relate to the intensification of oxidative stress.

### Ionomycin triggers endoplasmic reticulum stress and reactive oxygen species burst via calcium overload

**Figure 4:**
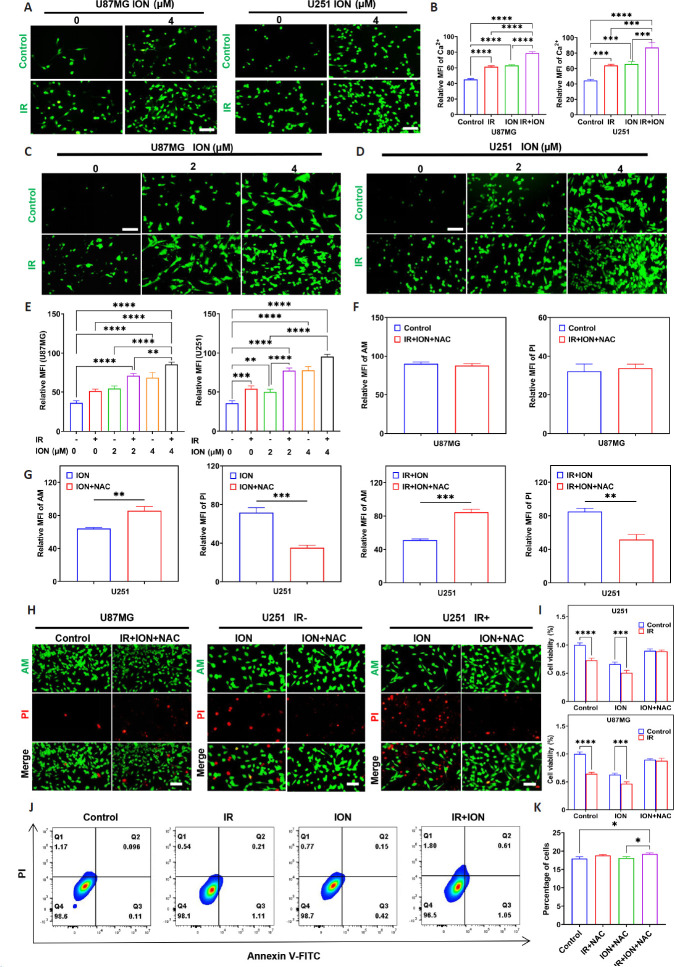
ION amplifies ROS levels to enhance IR by disrupting calcium homeostasis and the ERS pathway. (A) Calcium levels in U87MG and U251 cells were measured using the Fluo-4 fluorescent probe. Calcium levels increased in a dose-dependent manner with ION in both cell lines, and the intracellular fluorescence intensity was enhanced in the IR + ION group. (B) Quantitative analysis of calcium levels. (C–E) Quantitative analysis of intracellular ROS levels in U87MG and U251 cells. The intracellular ROS level in the IR + ION group was significantly enhanced and increased in a dose-dependent manner with ION. (F–I) Cell viability was assessed using live/dead cell staining (F–H) and the CCK-8 assay (I) in U87MG and U251 cells. The concentration of ION used was 4 μM. (J, K) The effect of NAC on ION-radiotherapy-induced apoptosis in U87MG cells was quantitatively analyzed using flow cytometry (Annexin V/PI staining). In all experiments involving the ROS scavenger NAC, the concentration used was 10 mM, and the pretreatment duration was 30 minutes. Scale bars: 200 µm. Data are expressed as mean ± SD (*n* = 3). *P < 0.05, **P < 0.01, ***P < 0.001, ****P < 0.0001 (one-way analysis of variance followed by Dunnett’s test (B, E, I, K) or unpaired *t*-test (F, G)). CCK-8: Cell counting kit-8; ERS: endoplasmic reticulum stress; ION: ionomycin; IR: ionizing radiation; MFI: mean fluorescence intensity; NAC: N-acetyl-L-cysteine; PI: propidium iodide; ROS: reactive oxygen species.

To be able to explain the mechanism, the ION effect on intracellular calcium ions was first investigated. The result of Fluo-4 fluorescence detection revealed that the combination treatment provoked considerable synergistic signal increase of calcium ions (**Figure 4A** and **B**), which revealed effective induction of sustained calcium overload by the combined approach. The result of the Western blotting indicated that treatment with ION alone, as well as the combination treatment, both had a significant upregulation effect on p-eIF2α and the ERS marker protein GRP78 (**Additional Figure 2A–D**). Intervention with the PERK pathway inhibitor ISRIB did not reduce p-eIF2α levels, consistent with its mechanism as an integrated stress response inhibitor. Since it is known that the signaling of calcium overload, ERS, and oxidative stress relies on tight signaling,^24^ we also explored the impact of ION on the intracellular ROS levels. The staining of DCFH-DA showed that ION caused a potent dose-dependent burst of ROS (**Figure 4C–E**). Besides, cell viability, necrosis, and apoptosis analyses indicated by combined IR and ION treatment suggested that the effect on radiosensitization was significantly reduced by pathway inhibition with NAC (**Figure 4F–K**). These results collectively indicate that ION, by inducing calcium overload, specifically activates the ERS pathway and simultaneously triggers significant oxidative stress, thereby providing the key initiating signal for synergistic promotion of apoptosis with IR.

### Ionomycin synergizes with radiotherapy to activate the reactive oxygen species–associated pro-apoptotic axis

**Figure 5:**
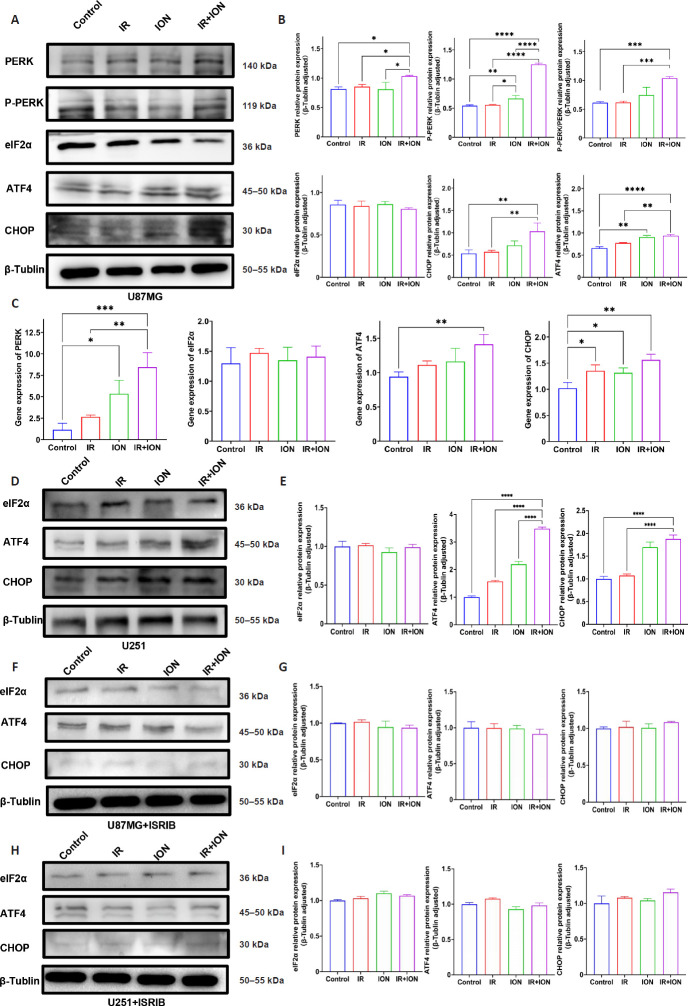
ION combined with IR activates the PERK-eIF2α-ATF4-CHOP axis. (A, B) Expression of key proteins in the PERK-eIF2α-ATF4-CHOP pathway in U87MG cells was quantified by Western blot assay. (C) mRNA expression changes of PERK, eIF2α, ATF4, and CHOP in U87MG cells were quantified by qPCR. (D, E) Expression of proteins in PERK-eIF2α-ATF4-CHOP pathway in U251 cells was quantified by western blot assay. (F–I) Expression of downstream proteins in U87MG and U251 cells was detected by Western blot assay. Data are expressed as mean ± SD (*n* = 3). *P < 0.05, **P < 0.01, ***P < 0.001, ****P < 0.0001 (one-way analysis of variance followed by Dunnett’s test). ATF4: Activating transcription factor 4; CHOP: C/EBP homologous protein; eIF2α: eukaryotic initiation factor 2α; ION: ionomycin; IR: radiotherapy; ISRIB: a PERK pathway inhibitor; PERK: protein kinase R-like endoplasmic reticulum kinase; qPCR: quantitative polymerase chain reaction.

Western blot and quantitative polymerase chain reaction results showed that when IR or ION was administered in U87MG cells, the expression of ATF4 and CHOP was moderately induced, whereas the synergistic and drastic increase of their expression occurred when the IR and ION were combined (**Figure 5A–C** and **Additional Figure 3**). The tendencies were also confirmed in U251 cells (**Figure 5D** and **E**). Interestingly, strong oxidative stress is also an important synergistic condition that leads to the complete activation of the PERK pathway and results in the mediation of apoptosis by the effect of CHOP. The ROS outburst activated by the combination therapy may have been a significant driving factor in this radical activation of the signaling axis. PERK downstream inhibition with ISRIB was effective in preventing the increase in the proteins of ATF4 and CHOP introduced by IR and ION (**Figure 3F–I**). These findings suggest that ION and IR are synergistic in the activation of the PERK-eIF2α-ATF4-CHOP signaling pathway, and the activation of the pathway is strongly connected to the above burst of ROS.

### Ionomycin combined with radiotherapy inhibits tumor growth *in vivo* by enhancing oxidative stress and apoptosis

**Figure 6:**
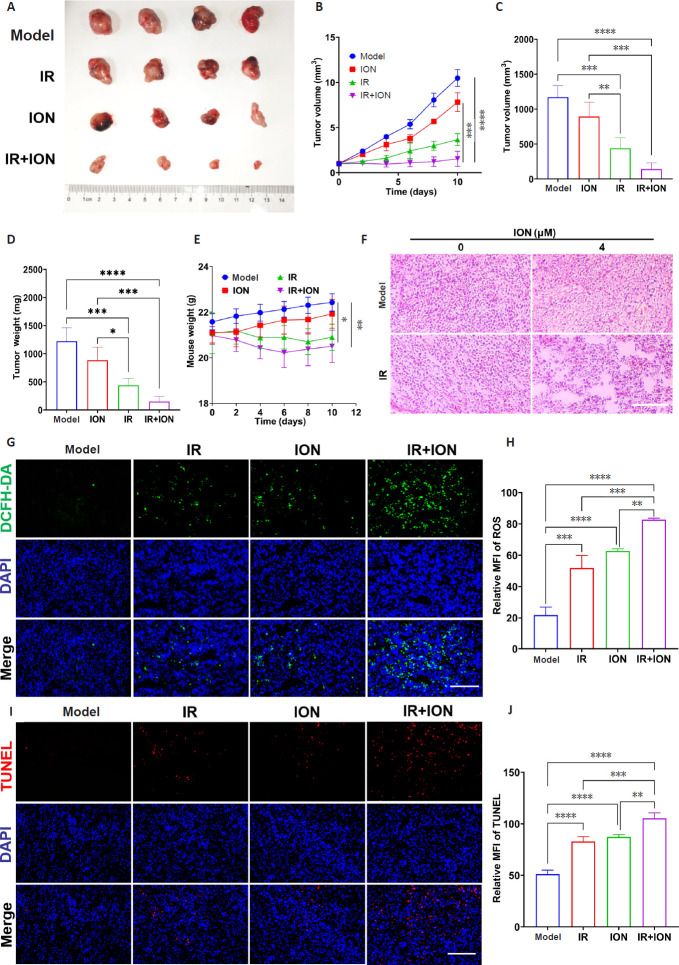
ION combined with IR inhibits GBM growth and induces apoptosis in vivo. (A) Representative images of tumors. (B-E) Tumor volume growth curves (B), relative tumor volume (C), final tumor weight (D), and mouse body weight (E). (F) H&E staining of tumor tissue sections. Compared with the other groups, tumor tissue in the IR + ION group exhibited more pronounced structural disruption and cellular necrosis. (G, H) ROS levels in tumor tissues were detected by DCFH-DA fluorescent probe staining (G) and quantified (H). The ROS fluorescence signal in the tumor tissue of the IR + ION group was significantly stronger than in the other groups. (I, J) Apoptosis in tumor tissues was detected by TUNEL staining (I) and quantified (J). The number of apoptosis-positive cells in the IR + ION group was significantly higher than those in other groups. Scale bars: 200 µm. Data are expressed as mean ± SD (*n* = 4/group). *P < 0.05, **P < 0.01, ***P < 0.001, ****P < 0.0001 (one-way analysis of variance followed by Dunnett’s test). DAPI: 4′,6-Diamidino-2-phenylindole; DCFH-DA: 2’,7-dichlorodihydrofluorescein diacetate; GBM: glioblastoma; H&E: hematoxylin and eosin; ION: ionomycin; IR: radiotherapy; MFI: mean fluorescence intensity; ROS: reactive oxygen species; TUNEL: terminal transferase dUTP nick end labeling staining.

In the U87MG subcutaneous xenograft model in nude mice, the IR and ION combination therapy indicated the strongest tumor growth inhibitory effect (**Figure 6A**). Compared with any of the monotherapy groups, there was also a significant reduction in tumor volume and weight, and no significant decrease in body weight was observed in the mice, indicating that ION has minimal toxic effects on mice (**Figure 6B–E**). The IR + ION group showed the most drastic destruction of tumor tissue, which is reflected visually in hematoxylin and eosin staining (**Figure 6F**). Mechanically, only minor increases in intratumoral ROS levels were observed during monotherapy, whereas the combination therapy really did lead to high levels of ROS signals (**Figure 6G** and **H**). Terminal transferase dUTP nick end labeling staining was consistently used to determine the percentage of apoptotic cells under the different treatment options; consistently, the IR + ION group significantly boosted the percentage of apoptotic cells (**Figure 6I** and **J**). These *in vivo* experiments also affirm that ION has a valid potential to regulate tumor development through IR as a synergizing agent by worsening oxidative stress and ERS.

## Discussion

For GBM, recurrence rates remain extremely high even after surgical intervention, making IR a crucial component of its overall treatment regimen. Nevertheless, the resistance of the tumor itself tends to cause the suboptimal outcome.^25^,^26^,^27^,^28^ Thus, it is important to seek synergistic mechanisms that can be actively applied to increase IR sensitivity to increase prognosis. Recent reports suggest that ERS and the resultant unfolded protein response may contribute to the fate of cells following IR, and IR-induced ROS is one of the mediators of it.^29^,^30^,^31^,^32^,^33^ It is suggested in this paper that through calcium homeostasis-ERS-ROS axis intervention with calcium ionophore ION, radiation-induced adaptive stress can turn into a fatal response. Mechanistically, irreversible calcium overload is initially caused by ION, which leads to the disruption of endoplasmic reticulum homeostasis, causing ERS.^34^,^35^,^36^ This further leads to mitochondrial dysfunction and enormous production of ROS. The acutely high rates of ROS at that moment reinforce the damage of the endoplasmic reticulum, and form the vicious cycle ION-ERS-ROS-ERS.^37^ This cycle and particularly activates the PERK-eIF2al-ATF4-CHOP pro-apoptotic axis, therefore, overcoming GBM cell resistance to IR.

IR may cause ERS, in turn, through DNA damage and ROS, but as an adaptive response, its level is not usually high enough to cause a large-scale death of tumor cells. Tumor cells can counteract therapy by activating pro-survival unfolded protein response branches (e.g., via epidermal growth factor receptor overexpression), enhancing SERCA function, and upregulating antioxidant defenses to clear ROS, thereby restoring homeostasis and achieving self-repair.^38^,^39^,^40^,^41^ To break this resistance, we introduced ION to disrupt calcium homeostasis and overwhelm cellular antioxidant capacity. Our results show that ION, combined with IR, produces significant synergistic anti-tumor effects both *in vitro* and *in vivo*, primarily inducing apoptosis. This effect was markedly attenuated by the ERS inhibitor ISRIB and accompanied by a ROS surge, confirming its dependence on the co-activation of ERS and oxidative stress pathways.

Calcium overload is a typical activator of ERS, as well as a non-negotiable activator of mitochondrial ROS synthesis.^42^ Being one of the primary cofactors of protein folding, the extreme disturbance of calcium homeostasis itself is an efficient ERS stimulator and a major control hub of mitochondrial ROS production.^43^,^44^ Our study delineates that the ION-mediated calcium overload potently and specifically activates the PERK-eIF2α axis. This giant increase in ROS generation is not a comorbid situation but rather an essential positive feedback loop that initiates and contributes to the sustainability of this pathway. It oxidatively modifies and stabilizes pro-apoptotic proteins such as CHOP directly.^45^,^46^ These results support that IR is the first sensitizing factor (ERS/ROS/transcriptional priming), and ION-induced irreversible calcium overload causes the vicious cycle. This subsequent ROS storm is the ultimate second signal, beyond the apoptotic threshold of cellular stress that results in the extreme expression of CHOP; the ROS-ERS cycle is the amplification engine of it.^47^ Through pharmacological regulation of calcium homeostasis by ION in order to regulate the ROS-ERS balance, this study provides an experimental approach to transform adaptive unfolded protein response signals into lethal ERS, offering new avenues to overcome SERCA-mediated repair and antioxidant defenses.

In addition to ERS/ROS-dependent apoptosis, ION can potentially work in synergy through its other functions (e.g., cell cycle modulation or ferroptosis induction), which is worth exploring further. In addition, there are some intrinsic disadvantages in the subcutaneous model to imitate the microenvironment in the intracranial sense. Future studies need to validate the efficacy of this combined strategy in orthotopic tumor models, with the aim of achieving brain-targeted drug delivery in clinical translation.

In summary, this study is innovative as it suggests an approach to increase the GBM radiosensitivity via the targeting of the calcium homeostasis-ERS-ROS axis. The mechanism involves ION-induced calcium overload triggering ERS, which subsequently causes mitochondrial ROS burst. The high ROS levels further aggravate endoplasmic reticulum damage, forming a self-reinforcing ION-ERS-ROS-ERS cycle. This process transforms repairable stress into an irreversible fatal response and greatly increases the effect of IR through the activation of the PERK-eIF2 -ATF4-CHOP pro-apoptotic cascade.

## Additional files:

***Additional Table 1:*** *qPCR primer sequences.*

***Additional Figure 1:*** *ION combined with IR enhances anti-tumor effects in vitro.*

Additional Figure 1 ION combined with IR enhances anti-tumor effects *in vitro*.
(A) Effects of ION on U87MG and U251 cell viability were assessed by CCK-8 assay. (B) The IC_50_ of ION was determined to be approximately 4.163 μM. (C) Clonogenic capacity of U87MG cells. Compared with the control group, the ION treatment group exhibited reduced clonogenic capacity; furthermore, following IR + ION therapy, the higher the radiation dose, the stronger the inhibitory effect on proliferation. (D) Proliferation of U87MG cells were measured over 3 days, and the dose-dependent effect of radiation on cell proliferation was evaluated. Data are expressed as mean ± SD (*n* = 3). ^*^P < 0.05, ^**^P < 0.01, ^***^P < 0.001, ^****^P < 0.0001 (one-way analysis of variance followed by Dunnett's test (A) or unpaired *t*-test (D)). CCK-8: Cell counting kit-8; IC_50_: half-maximal inhibitory concentration; ION: ionomycin; IR: ionizing radiation.

***Additional Figure 2:*** *ION combined with IR triggers ERS and ROS burst via calcium overload.*

Additional Figure 2 ION combined with IR triggers ERS and ROS burst via calcium overload.
(A, C) Western blot detection and quantification of ERS-labeled protein expression in U87MG and U251 cells. (B, D) Quantitative analysis of the expression levels of p-eIF2α, and GRP78 following treatment with the stress response inhibitor ISRIB. Data are expressed as means ± SD (*n* = 3). ^***^P < 0.001, ^****^P < 0.0001 (one-way analysis of variance followed by Dunnett's test). ERS: Endoplasmic reticulum stress; GRP78: glucose-regulated protein 78; ION: ionomycin; IR: ionizing radiation; ISRIB: a PERK pathway inhibitor; p-eIF2α: phosphorylated eukaryotic initiation factor 2α; PERK: protein kinase R-like endoplasmic reticulum kinase; ROS: reactive oxygen species.

***Additional Figure 3:*** *ION combined with IR activates the eIF2α in U87MG cells.*

Additional Figure 3 ION combined with IR activates the eIF2α in U87MG cells.
(A) The p-eIF2α/eIF2α was assessed by Western blot assay. Data are expressed as mean ± SD (*n* = 3). ^**^P < 0.01 (oneway analysis of variance followed by Dunnett's test). eIF2α: Eukaryotic initiation factor 2α; ION: ionomycin; IR: ionizing radiation; p-eIF2α: phosphorylated eukaryotic initiation factor 2α.

## Data Availability

*All data generated or analyzed in this study are included in this published article and its additional files.*
